# Performance Evaluation of Multiple Ultrasonographical Methods for the Detection of Primary Sjögren’s Syndrome

**DOI:** 10.3389/fimmu.2021.777322

**Published:** 2021-11-22

**Authors:** Shihao Xu, Jing Luo, Chengwei Zhu, Jiachun Jiang, Hui Cheng, Ping Wang, Jingwei Hong, Jinxia Fang, Jingjing Pan, Matthew A. Brown, Xiaochun Zhu, Xiaobing Wang

**Affiliations:** ^1^ Department of Ultrasonography, The First Affiliated Hospital of Wenzhou Medical University, Wenzhou, China; ^2^ Beijing Tsinghua Changgung Hospital, School of Clinical Medicine, Tsinghua University, Beijing, China; ^3^ Department of Rheumatology, The First Affiliated Hospital of Wenzhou Medical University, Wenzhou, China; ^4^ Department of Rheumatology, Taizhou Hospital of Zhejiang University, Linhai, China; ^5^ Department of Laboratory Medicine, The First Affiliated Hospital of Wenzhou Medical University, Wenzhou, China; ^6^ Department of Medicine, Guy’s and St Thomas’ Hospital NHS Trust and King’s College London NIHR Biomedical Research Centre, London, United Kingdom; ^7^ Department of Rheumatology and Immunology, Shanghai Changzheng Hospital, Second Affiliated Hospital of Naval Medical University, Shanghai, China

**Keywords:** primary Sjögren’s syndrome, grayscale ultrasonography, color Doppler sonography, contrast-enhanced ultrasonography, diagnostic model

## Abstract

Major salivary gland ultrasonography (SGUS) is increasingly being recognized as having critical roles in differentiating primary Sjögren’s syndrome (pSS) from other connective tissue disorders. Contrast-enhanced ultrasonography (CEUS) has been reported to evaluate microvascularity of lesions in different tissues with objective angiographic index, eliminating the observer-dependent defect of ultrasonography. However, there are few relevant studies concentrating on the application of CEUS in the diagnosis and assessment for pSS, and their clinical utility prospect remains uncertain. In this study, a total of 227 eligible patients were enrolled, including 161 pSS and 66 non-pSS patients with comprehensive ultrasonographic evaluation of the parotid and submandibular glands, including grayscale ultrasonography, color Doppler sonography (CDS), and CEUS. Compared with non-pSS, pSS patients had significantly higher grayscale ultrasound (US) scores and CDS blood grades in the parotid gland and significantly higher grayscale US and CEUS scores in the submandibular glands. Diagnostic model combining ultrasonographic signatures, anti-SSA/Ro60, and keratoconjunctivitis sicca (KCS) tests showed a remarkable discrimination [mean area under the curve (AUC)0.963 in submandibular glands and 0.934 in parotid glands] for pSS, and the nomogram provided excellent prediction accuracy and good calibration in individualized prediction of pSS. A combination of multiple ultrasonographical examinations of the major salivary glands (SGs) is a promising technique that may be used as a practical alternative to minor SG biopsy in the detection of pSS.

## Introduction

Primary Sjögren’s syndrome (pSS) is a common chronic systemic autoimmune disease characterized by focal lymphocytic infiltration of the exocrine glands, especially in the salivary and tear glands, mainly presenting as xerostomia, xerophthalmia, and other extraglandular involvements (1). With a population prevalence of 0.05%–0.6% and a nearly 14:1 female/male ratio, pSS mostly affects middle-aged females between 30 and 50 years at the time of diagnosis (2, 3). Detection of pSS is a challenge due to the complex nature and heterogeneity of the disease and no gold standard test. An average of 7 years is required from onset of symptoms to final diagnosis (4). Current classification criteria (5, 6) have greatly improved this situation, with histopathology of minor salivary gland biopsy (MSGB) a dominant factor. However, the invasive nature of MSGB, with high risks of several complications such as persistent sensory deprivation caused by local nerve injury and pyogenic granuloma (7, 8), means it is still not accepted by all patients. Hence, there is an urgent unmet need for improved and non-invasive methods to facilitate the detection of pSS.

As a low-cost, non-invasive, repeatable, and effective technique without ionizing radiation, major salivary gland ultrasonography (SGUS) assists in differentiating pSS from non-immune-mediated sicca syndrome and other connective tissue disorders, with good sensitivity and high specificity (9–12). Recently, massive studies have demonstrated that grayscale ultrasound (US) could clearly display a subtle sonographic abnormality of the salivary glands (SGs) in pSS, including the glandular border, echostructure, and acoustic effects (13, 14). Furthermore, color Doppler sonography (CDS) could reflect changes in glandular hemodynamics by evaluating microvascular vascularity index values in pSS (15). Although ultrasonographical techniques are highly operator- and observer-dependent, various scoring systems, including De Vita score (16), Hocevar score (17), Salaffi score (18), Jousse-Joulin score (10), and OMERACT semiquantitative score (19), have been used to evaluate the typical SGUS changes of pSS, and many efforts have been made in the assessment of consensus and reliability of these scores for pSS, even in European Union (EU)-funded projects (20). However, their clinical utility prospect for the detection of pSS remains to be further investigated (21).

Contrast-enhanced ultrasonography (CEUS) has been reported to evaluate microvascularity of lesions in different tissues with objective angiographic index, eliminating the observer-dependent defect of US. CEUS has been applied to display the different characterizations of benign and malignant tumors and further estimate the therapeutic efficacy after chemotherapy, especially in hepatocellular carcinoma (22, 23). In addition, it was demonstrated that CEUS may play a significant role in detection of cancer in other non-hepatic organs (24). Moreover, several studies found CEUS valuable to evaluate dynamic microcirculation for localization of pathological glands, including the parathyroid (25) and SGs, especially for the differential diagnosis of benign and malignant lesions (26). Giuseppetti et al. (27) also demonstrated that the time–intensity curves (TICs) of contrast-enhanced US could provide useful information for sicca characterization and severity assessment. However, there are few relevant studies concentrating on the application of CEUS in the diagnosis of and assessment for pSS.

Currently, no classification criteria for pSS include US of the SGs, despite the increasing evidence that this technique might add diagnostic value for pSS (28). In this study, we explored the feasibility of using routine US techniques (grayscale and CDS), and first applied CEUS, to evaluate the structural and microvascular lesions of the parotid and submandibular glands. Moreover, a clinical prediction model was constructed, combined with US techniques and serological index, without an MSGB test.

## Materials and Methods

### Patients and Data Preparation

A total of 250 consecutive clinically suspected pSS candidates with xerostomia and/or xerophthalmia were enrolled from the Rheumatology Department of the First Affiliated Hospital of Wenzhou Medical University between January 1, 2019, and July 1, 2020. Fifteen candidates with severe cardiovascular and pulmonary diseases and eight with unstable vital signs or regional SG nodules or tumors, were excluded. The remaining 227 patients, all older than 18 years, underwent a comprehensive workup including physical examination, serological testing, MSGB, keratoconjunctivitis sicca (KCS) detection, and US examination of the parotid and submandibular glands. The KCS detection was performed by an experienced ophthalmologist including ocular staining, Schirmer’s test, and breakup time of tear film (BUT). These patients were subsequently divided into 161 pSS and 66 non-pSS subgroups based on the 2016 American College of Rheumatology (ACR)/European League Against Rheumatism (EULAR) (6) or 2012 ACR criteria (5). Detailed clinical information was recorded, and EULAR Sjögren’s Syndrome Disease Activity Index (ESSDAI) scores based on 2010 EULAR criteria (29) were used to reflect disease activity and systemic involvement. This study was approved by the ethics committee of the First Affiliated Hospital of Wenzhou Medical University, and written informed consent was received from all participants.

### Assessment of Multimodal Ultrasound

Examinations of US were performed by two experienced sonographers. The LOGIQ E9 ultrasonographic scanner (GE Healthcare, Madison, WI, USA) equipped with an ML6-15-D linear array probe (6–15 MHz, GE Healthcare, Madison, WI, USA) was used to perform gray scale and CDS. The patient was placed in a supine position with overextension of the neck and the head turned to the opposite side. The bilateral parotid and submandibular glands were examined in the longitudinal and transverse planes. The semiquantitative SGUS score (0–16) was acquired by summing scores (0–4) of each parotid and submandibular gland according to the criteria of De Vita et al. (16). Intensity of blood flow in the SGs was evaluated by CDS with 7.5 MHz and classified as followsgrade 0, absence of blood flow; grade I, focal blood flow; grade II, marked blood flow (26).

A second-generation US contrast medium (sulfur hexafluoride, SonoVue; Bracco, Milan, Italy) was intravenously administered at a dose of 4.8 ml as a bolus and was subsequently flushed with 10 ml of saline. The examination was documented with a 90-s clip, starting at the beginning of the bolus injection. Data acquisition lasted approximately 10 min per patient, and kinetic analysis of the CEUS parameter was performed with dedicated software (VueBox; Bracco Suisse SA, Geneva, Switzerland). CEUS parameters were recorded and analyzed frame by frame using the fitting TIC quantification software by a well-trained sonographer with more than 5 years’ experience who was blinded to the results of diagnosis and disease status. A region of interest (ROI) was manually drawn on the target gland, and the following quantitative micro perfusion parameters were obtained with the fitting TIC that was constructed for the ROIarea under the curve (AUC) (Area), maximum ascending gradient (Grad), arrive time (Atm), time to peak (TtoP), peak intensity (PI), and intensity difference (ID).

### Association Between Clinical Features and Ultrasound Characteristics

We performed a comparison of multiple US indices between pSS and non-pSS subgroups with Wilcoxon or chi-square test. Moreover, we further grouped the pSS and non-pSS cohorts into opposing groups based on different clinical phenotypes to explore their US characteristics. For example, based on the ESSDAI scores, the pSS cohorts were divided into I~III groups (I/mild, 0–4; II/medium, 5–13; III/severe, ≥14), and pathologically positive groups were identified as the patients with focus score ≥1 foci/4 mm^2^ in MSGB. Besides, in pSS patients, we also analyzed the lymphadenopathy and glandular ESSDAI domains (no, low, moderate) with the different US techniques. In addition, the relationship between CEUS signatures and pSS was investigated using a univariate logistic analysis to preliminarily screen pSS-associated indices, followed by further multivariate logistic regression. Candidate parameters with significant statistical values (p < 0.05) were selected to identify representative CEUS scores used for the establishment of a diagnostic model. The CEUS signatures of each patient were determined using:


CEUS score=val(Signature1)∗β1+val(Signature2)∗β2+⋯+val(Signature n)∗βn+intercept value

where “val” represents values of CEUS parameters, and “β” represents the regression coefficient. In this study, six quantitative CEUS parameters of the parotid and submandibular glands were chosen to conduct univariate logistic regression analysis, including TtoP, Grad, Area, PI, Atm, and ID, and four submandibular CEUS parameters (Area, Grad, PI, and ID) were further screened for subsequent multivariable logistic regression analysis (**Supplementary Table S1**). Ultimately, submandibular Grad, PI, and ID were chosen to identify the final CEUS score as followsCEUS score = 13.087-0.003*Grad-0.811*ID-0.164PI.

### Construction and Evaluation of Diagnostic Model for Primary Sjögren’s Syndrome

The randomForest package (30) was used to conduct random forest models with 100 runs of cross-validation, which predicted pSS based on multiple ultrasonographic indices and other key clinical features, including anti-SSA/Ro60, ocular staining, and Schirmer’s test, with or without MSGB. For each cross-validation, the 227 patients were randomly separated into a training set (n = 159) and a validation set (n = 68) according to the 7:3 ratio. Receiver operating characteristic (ROC) curves and significant indices including optimal cutoff point, AUC, sensitivity, and specificity were identified using the “ROCR” package to evaluate the efficiency of models (31). Furthermore, the probability of disease (POD) value was calculated to estimate accuracy, and the mean decrease of Gini coefficient was used to assess the impact of each variable in the random forest models. We also built different diagnostic models based on individual ultrasonographic signatures and clinical features to make a direct comparison of the discriminatory power using AUC value.

Two submaxillary US signatures (including grayscale US and CEUS scores) and classical clinical diagnostic indices (including anti-SSA/Ro60 and KCS examination) with significant diagnostic values (p < 0.05) were chosen to construct convenient and efficient nomograms for individualized prediction of pSS by using rms package (32). Significant parotid US parameters (including grayscale US scores and CDS blood grades) were also chosen to construct effective nomograms combined with the above clinical diagnostic index. Calibration curves of the nomogram in its corresponding training and validation sets were plotted to compare prediction and real observation in diagnosis of pSS through a bootstrapping method with 1,000 resamples. The C-index was also measured to evaluate the discriminatory power of the model. The clinical usefulness of the diagnostic nomogram model was determined by the decision curve analysis (DCA) after calculating the net benefits for patients at different risk threshold probabilities (33). Moreover, according to the DCA, clinical impact curves (CICs) were plotted to help us more intuitively assess the nomogram model’s diagnostic value for pSS.

### Statistical Analysis

All statistical analyses were performed in R software (version 4.0.1, https://www.r-project.org/). Continuous and classified variables were presented as mean ± standard deviation and number (percentages), respectively. Wilcoxon signed-rank test was used to compare continuous variables, and chi-square test was applied to compare categorical variables. The two-tailed p-value <0.05 was considered statistically significant.

## Results

### Clinical Characteristics of Study Population

**Figure 1:**
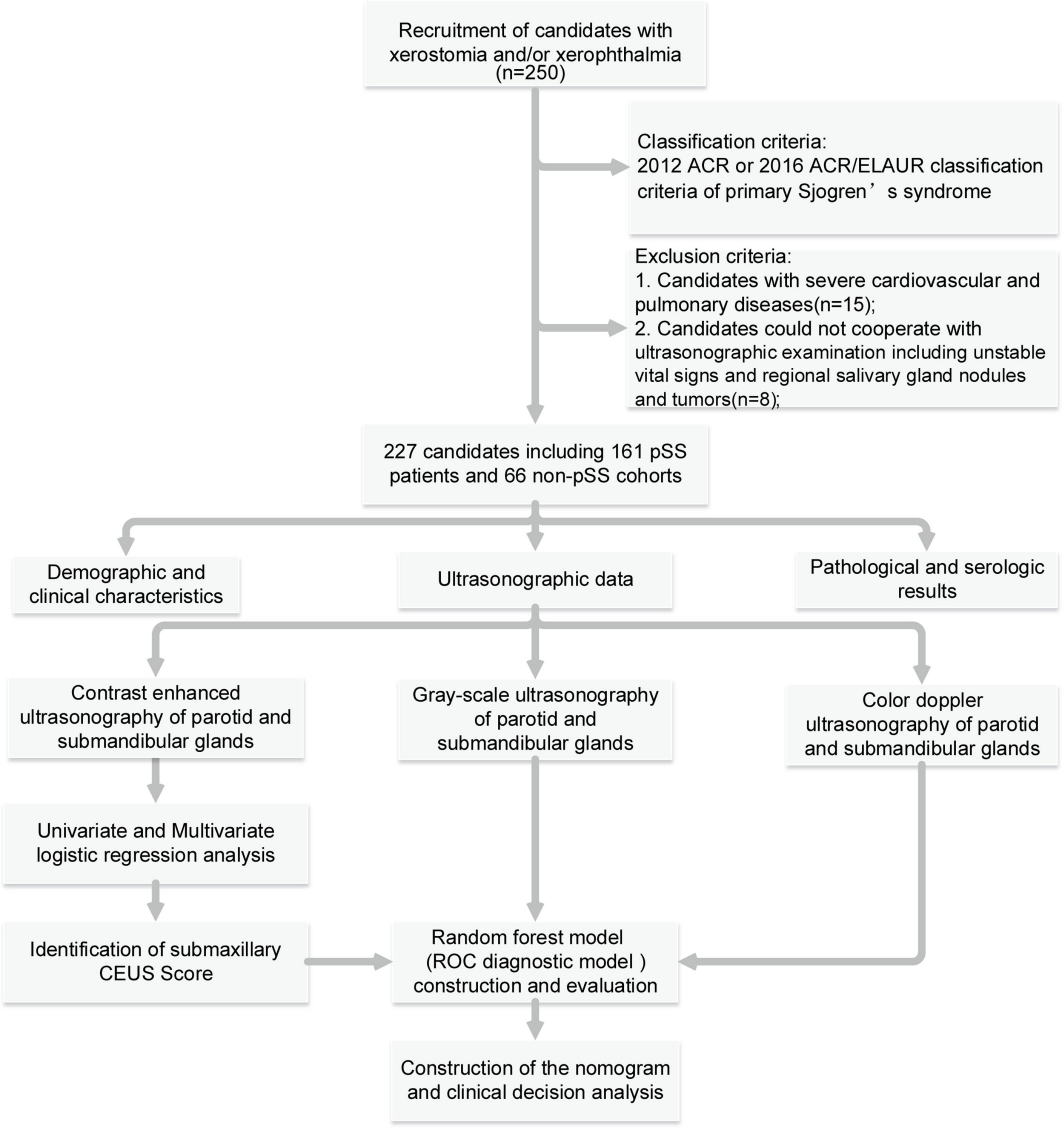
Summary and description of the study workflow.

The overall workflow of the study is shown in **Figure 1**, and the clinical characteristics of the study cohorts are displayed in **Table 1**. In the pSS group, 149 (92.50%) patients were female and the average age was 48.07 years, ranging from 36.00–60.14, with a mean ESSDAI score of 8 (range, 4–15). There was no significant difference between the pSS and non-pSS groups in age and gender. pSS patients had similar prevalence of xerostomia (51.60% *vs*. 51.50%) and xerophthalmia (43.50% *vs*. 37.90%) but significantly higher rates of MSGB positivity (75.80% *vs*. 18.20%), KCS positivity (78.90% *vs*. 43.90%), antinuclear antibody (ANA) positivity (95.00% *vs*. 68.20%), anti-SSA/Ro60 positivity (82.60% *vs*. 13.60%), anti-SSA/Ro52 positivity (68.90% *vs*. 24.20%), RF positivity (28% *vs*. 18.20%), hypergamma-globulinemia (60.90% *vs*. 24.30%), and higher erythrocyte sedimentation rate levels (25.75 ± 21.60 mm/h *vs*. 13.64 ± 11.83 mm/h) compared with the non-pSS subgroup (p < 0.05 for all between-group comparisons).

**Table 1:** Demographic and clinical characteristics of pSS and non-pSS patients.

Characteristic	pSS (n = 161)	non-pSS (n = 66)	p*-*value
**Demographic characteristics**			
Age, y, mean ± SD	48.07 ± 12.07	48.23 ± 13.47	0.950
Sex, female, n (%)	149 (92.50%)	58 (87.90%)	0.260
**Key clinical features**			
Xerostomia, n (%)	83 (51.60%)	34 (51.50%)	0.996
Xerophthalmia, n (%)	70 (43.50%)	25 (37.90%)	0.437
**Laboratory findings:**			
MSGB, lymphocytic focus ≥1, n (%)	122 (75.80%)	12 (18.20%)	<0.001^***^
KCS, n (%)	127 (78.90%)	29 (43.90%)	<0.001^***^
ANA-positive (ANA > 1:100), n (%)	153 (95.00%)	45 (68.20%)	<0.001^***^
Anti-SSA/Ro60‐positive, n (%)	133 (82.60%)	9 (13.60%)	<0.001^***^
Anti-SSA/Ro52‐positive, n (%)	111 (68.90%)	16 (24.20%)	<0.001^***^
Anti-La/SSB‐positive, n (%)	65 (40.40%)	1 (1.50%)	<0.001^***^
Low C3 levels (<0.9 g/L), n (%)	38 (23.60%)	17 (25.80%)	0.731
Low C4 levels (<0.1 g/L), n (%)	10 (6.20%)	4 (6.10%)	0.966
Hypergammaglobulinemia (>16 g/L), n (%)	98 (60.90%)	16 (24.30%)	<0.001^***^
RF-positive, n (%)	45 (28.00%)	12 (18.20%)	0.123
ESR, mm/h, median (25%–75% RI)	20 (8-27.75)	11 (9-28)	<0.001^***^
ESSDAI score, median (25%–75% RI)	8 (4-15)	–	–

MSGB, minor salivary gland biopsy; KCS, keratoconjunctivitis sicca; ANA, antinuclear antibodies; RF, rheumatoid factor; ESR, erythrocyte sedimentation rate; ESSDAI, EULAR Sjogren’s Syndrome Disease Activity Index; RI, range interquartile. ***p < 0.001.

### Characteristics of Imaging Abnormalities in Primary Sjögren’s Syndrome

**Figure 2:**
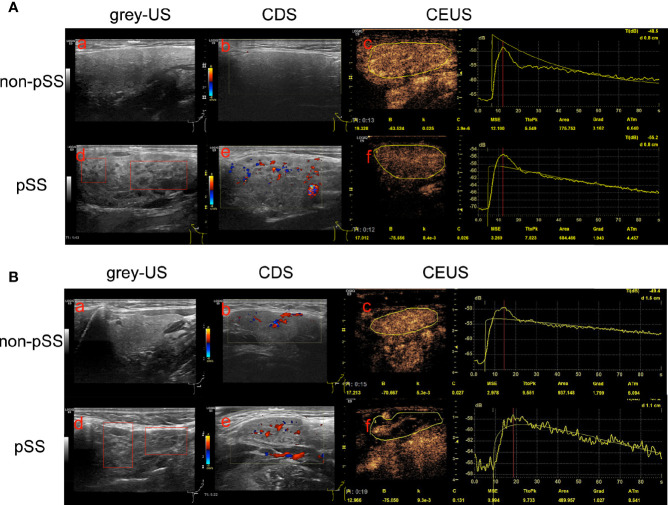
Multimodal ultrasonographic imaging characteristics of the parotid and submandibular glands between the primary Sjögren’s syndrome (pSS) and non-pSS subgroups. **(A)** Parotid glands: (a) homogeneous parenchyma without anechoic or hypoechoic areas in the non-pSS cohort; **(d)** diffuse inhomogeneity with anechoic or hypoechoic areas in pSS patients; CDS identified abundant hypervascularization in pSS cohorts **(e)** with poor vascularization in non-pSS groups **(b)**; **(c, f)** No echo-free areas and delay TtoP in the parotid glands of pSS compared with non-pSS cohorts. **(B)** Submandibular glands: (a) homogeneous parenchyma without anechoic or hypoechoic areas in the non-pSS cohort; **(d)** diffuse inhomogeneity with anechoic or hypoechoic areas in pSS patients; **(b, e)** abundant hypervascularization detected in both pSS and non-pSS patients; **(c, f)** massive echo-free areas without contrast enhancement and significant delay TtoP after contrast media injection in pSS patients compared with non-pSS cohorts. US, Ultrasonography; CDS, Color Doppler sonography; CEUS, Contrast-enhanced ultrasonography; pSS, primary Sjögren’s syndrome.

The multimodal US imaging characteristics are illustrated in **Figure 2**. The grayscale US exhibited homogeneous parenchyma in both the parotid and submandibular glands in the non-pSS cohort, while diffuse inhomogeneity with anechoic or hypoechoic areas was significantly detected in these glands in the pSS group (**Figures 2A, B-a, d**). Abundant hypervascularization was detected by CDS in the parotid and submandibular glands of pSS patients. Similar vascularization was found in the submandibular glands and less vascularization in the parotid glands of the non-pSS subgroup (**Figures 2A, B-b, e**). Furthermore, obvious larger echo-free areas without contrast enhancement and delayed peak time after contrast media injection were detected by CEUS in the submandibular glands, but not the parotid glands, of the pSS group (**Figures 2A, B-c, f**).

**Figure 3:**
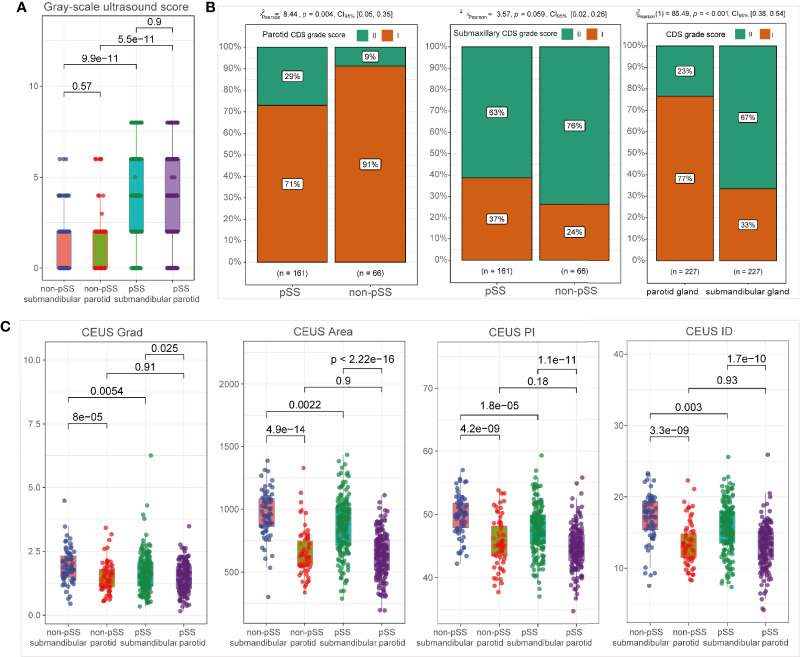
Ultrasonographic abnormalities in the salivary glands of primary Sjögren’s syndrome (pSS). Comparison of grayscale ultrasound (US) scores **(A)**; CDS grade scores **(B)**; and CEUS parameters **(C)** between subgroups, including pSS *vs*. non-pSS and parotid *vs*. submandibular glands.

The grayscale US scores of bilateral parotid and submandibular glands were significantly more elevated in the pSS subgroup than those in the non-pSS subgroup (p < 0.001) (**Figure 3A**). Regarding the blood intensity of the parotid glands detected by CDS, pSS patients demonstrated grade I changes (71% grade I, 29% grade II) more often compared with non-pSS patients (91% grade 1, 9% grade II; p = 0.004). However, there was no significant difference in the blood intensity of submandibular glands between pSS (grade I, 37% patients; grade II, 63% patients) and non-pSS subgroups (grade I, 24% patients; grade II, 76% patients) (p = 0.059) (**Figure 3B**). Notably, CEUS parameters, including Grad, Area, PI, and ID, were significantly decreased in the submandibular glands of pSS patients compared to non-pSS patients (p < 0.0001); however, no significant differences in CEUS parameters were found in the parotid glands between the two groups (**Figure 3C**, **Supplementary Figure S1A**).

### Ultrasound Differences Between the Parotid and Submandibular Salivary Glands

As shown in **Figure 3A**, there was no significant difference in grayscale US scores between the parotid gland and submandibular glands in neither pSS nor non-pSS groups. However, blood intensity detected by CDS was significantly higher in the submandibular gland than that in the parotid gland (**Figure 3B**; p = 0.001). According to CEUS indices, higher levels of Grad, Area, PI, and ID were detected in the submandibular glands compared to the parotid glands both in the pSS and non-pSS cohorts (**Figure 3C**), and there was no significant difference in TtoP and Atm (**Supplementary Figure S1A**). To further evaluate whether ultrasonographic scores of unilateral glands were sufficient for the application of ultrasonographic diagnosis in pSS, we also performed the comparison of grayscale US scores between left and right parotid and submandibular glands. No significant statistical difference was observed between the two sides of parotid and submandibular glands for grayscale US scores in both pSS and non-pSS subgroups (**Supplementary Figure S1B**).

### Association Between Ultrasonographic Features and Clinical Manifestations of Primary Sjögren’s Syndrome

**Figure 4:**
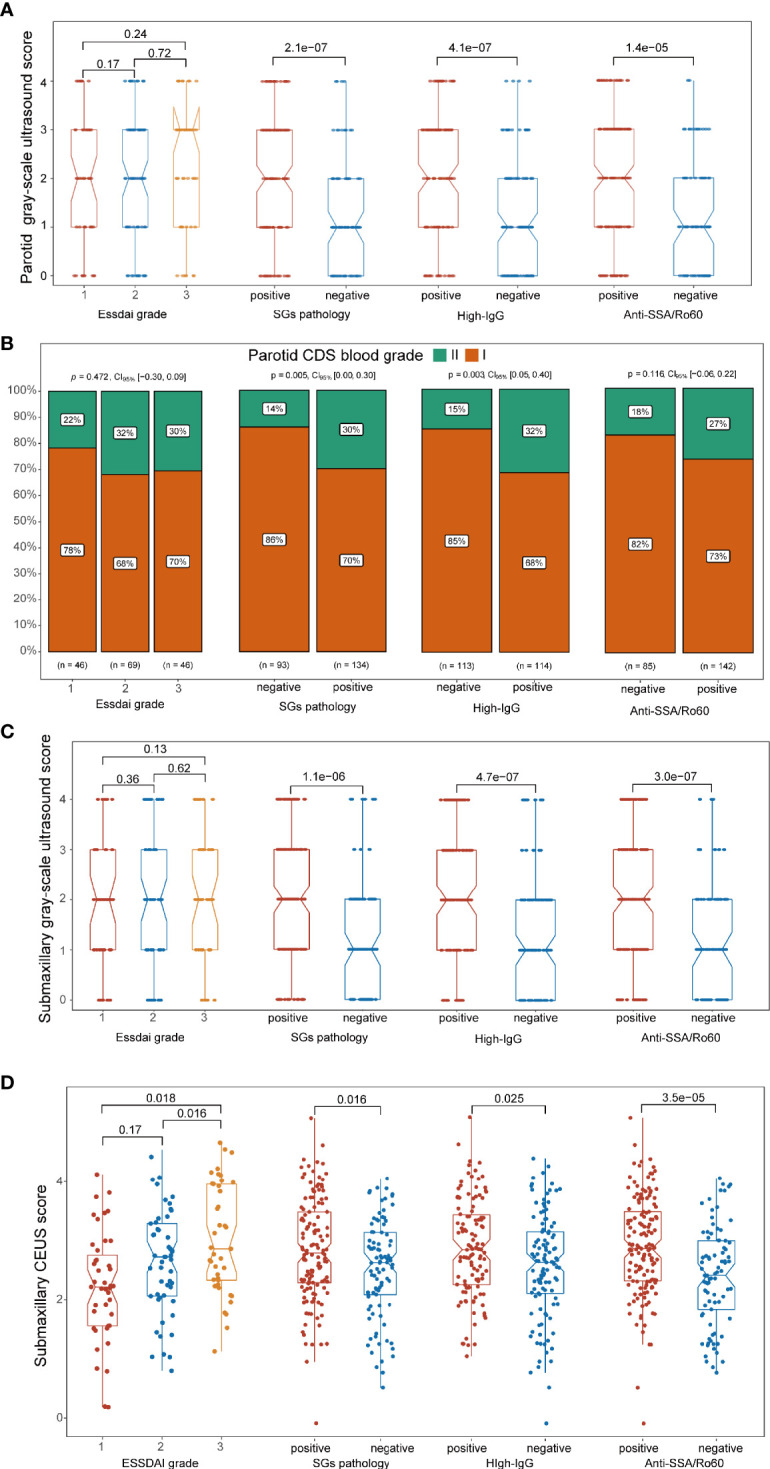
Association between ultrasonographic signatures and significant clinical phenotypes of primary Sjögren’s syndrome (pSS). **(A, C)** In both the parotid and submandibular glands, the grayscale ultrasound (US) scores were higher in pathologically positive, high IgG-positive, and anti-SSA/Ro60-positive groups (p < 0.001) than their corresponding groups, while there was no significant correlation in ESSDAI grade groups. **(B)** Higher rate of CDS grade II in the parotid glands was found in the pathologically positive group (p = 0.005) and high-IgG groups (p = 0.003), while there was no significant difference between subgroups in ESSDAI and anti-SSA/Ro60 phenotypes (p > 0.05). **(D)** Higher CEUS scores in submandibular glands were found in the pathologically positive group (p = 0.016), high-IgG group (p = 0.025), anti-SSA/Ro60-positive group (p < 0.001), and higher ESSDAI grades (p < 0.05).

To further investigate the correlation between ultrasonographic features and clinical manifestations of pSS, we divided the cohort into different subgroups based on the clinical manifestations and compared the ultrasonographic difference according to grayscale US scores, CDS blood grades, and CEUS scores. Both in parotid and submandibular glands, it turned out that the grayscale US scores were higher in SG pathology-positive patients, high-IgG level group, and anti-SSA/Ro60-positive groups than their corresponding control groups (p < 0.001), while there was no significant difference in the grayscale US scores among patients with different ESSDAI grades (**Figures 4A, C**). In addition, higher rate of CDS blood intensity grade II in parotid glands was found in pathologically positive group (p = 0.005) and high-IgG groups (p = 0.003), while there was no significant difference between subgroups for submandibular glands in neither ESSDAI nor anti-SSA/Ro60 phenotypes (p > 0.05; **Figure 4B**). Moreover, higher CEUS scores in submandibular glands were not only found in pathologically positive group (p = 0.016), high-IgG group (p = 0.025), and anti-SSA/Ro60-positive group (p < 0.001), but also in patients with higher ESSDAI grades (p < 0.05; **Figure 4D**). In addition, there was no significant statistical difference of CDS characteristics among distinct severities of both lymphadenopathy and glandular ESSDAI domain involvements. Interestingly, there was no significant difference in grayscale US score among different severities of lymphadenopathy ESSDAI domain involvements, while the grayscale US scores were higher in the moderate group than that in the group without glandular involvement in both parotid and submandibular glands (p = 0.023 and p = 0.038, respectively). Notably, as to glandular ESSDAI domain involvements, the CEUS scores were significantly positively associated with the severity of glandular abnormality, and there was no difference in lymphadenopathy ESSDAI domain involvements (**Supplementary Figures S1C, D**).

### Diagnostic Value of Ultrasound Signatures for Primary Sjögren’s Syndrome

**Figure 5:**
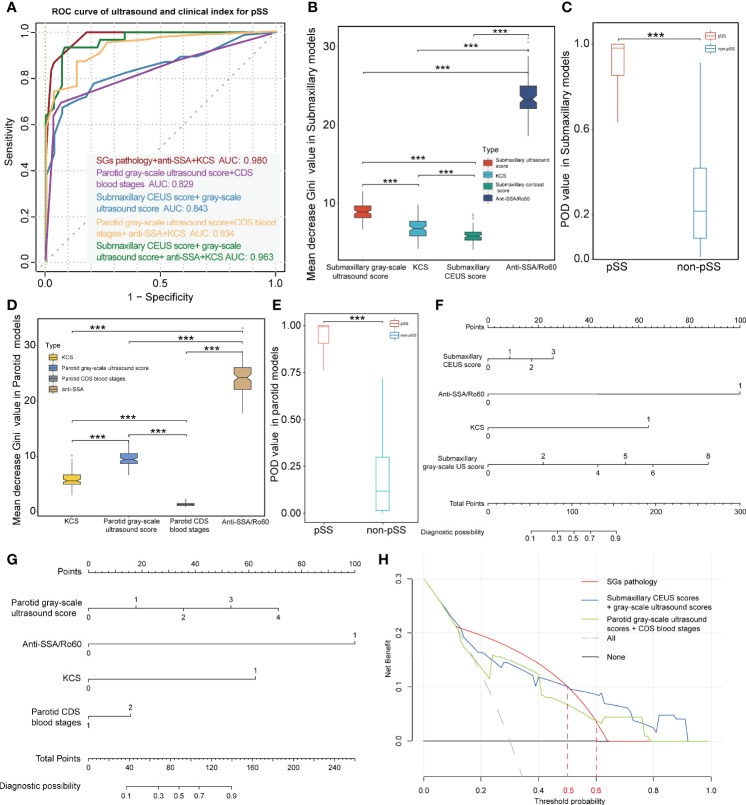
Diagnostic capacity of ultrasonographic signatures for primary Sjögren’s syndrome (pSS). **(A)** ROC curve of pSS prediction using the random forest models; red lines indicate the diagnostic capacity of clinical indices with MSGB (AUC 0.980); purple lines indicate the diagnostic capacity of the model with parotid grayscale ultrasound score and CDS blood stages (AUC 0.829); blue lines indicate the diagnostic capacity of the model with submaxillary CEUS score and grayscale ultrasound score (AUC 0.843); yellow lines indicate the diagnostic capacity of the model combining clinical indices and parotid ultrasonographic signatures without MSGB (AUC 0.934); and green lines indicate the diagnostic capacity of the model combining clinical indices and submaxillary ultrasonographic signatures without MSGB (AUC 0.963). **(B, D)** Mean decrease Gini coefficient represents the specific diagnostic capabilities of variables in the construction of the predicting model. Variable importance of ultrasonographic signatures is lower than anti-SSA/Ro60 positivity but significantly higher than KCS. **(C, E)** The combined models without MSGB reveal a high predictive accuracy with higher POD value for pSS in both the parotid and submandibular glands. Nomograms were developed to aid in predicting risk of pSS using the four prognostic factors in parotid **(F)** and submandibular glands **(G)**. **(H)** DCA reveals the threshold probability of a patient was >50%/>60%. Using submandibular/parotid ultrasonographic examination adds more net benefit than using pathological results of the salivary glands for the prediction of pSS. ***p < 0.001.

In the submandibular glands, we revealed that ultrasonographic signatures manifested a high discriminatory capability in distinguishing pSS from non-pSS, with a mean AUC value of 0.807 for grayscale US scores and 0.764 for CEUS scores. However, CDS blood grades displayed poor discriminative capability with a mean AUC value of 0.565 (**Table 2**). In contrast, grayscale US scores and CDS blood grades also performed a good discriminatory capability, with mean AUC values of 0.809 and 0.639, respectively, in the parotid glands. However, the discriminative capability of the US model was significantly increased when grayscale US scores were combined with CEUS scores in the submandibular glands (mean AUC = 0.843) and with CDS blood grades in the parotid glands (mean AUC = 0.829). Notably, using ultrasonographic signatures (grayscale US and CEUS scores in the parotid glands, grayscale US and CDS blood grades in the submandibular glands) to replace the SG pathology, combined with anti-SSA/Ro60 and KCS positivity phenotypes, the model still showed a high discriminative accuracy for pSS, with mean AUC values of 0.963 and 0.934, respectively (**Figure 5A**).

**Table 2:** Diagnostic models of parotid and submaxillary ultrasound and clinical index for pSS.

Variable	Optimal cutoff point	Area under curve (AUC)	Sensitivity	Specificity
Submaxillary CEUS score	0.025	0.764	0.639	0.774
Submaxillary grayscale ultrasound score	0.875	0.807	0.867	0.655
Submaxillary CDS blood grade	1.500	0.565	0.758	0.373
Submaxillary grayscale ultrasound score and CDS blood grade	0.035	0.804	0.583	0.900
Submaxillary CEUS score and grayscale ultrasound score	0.280	0.843	0.671	0.931
Parotid grayscale ultrasound score	0.840	0.809	0.931	0.671
Parotid CDS blood grade	0.005	0.639	0.345	0.933
Parotid grayscale ultrasound score and CDS blood grade	0.305	0.829	0.694	0.931
MSGB and anti-SSA/Ro60 and KCS	0.630	0.980	0.867	0.964
MSGB	0.670	0.888	0.852	0.923
Anti-SSA/Ro60	0.515	0.849	0.844	0.854
KCS	0.960	0.792	0.758	0.827
Submaxillary CEUS score and grayscale ultrasound score and anti-SSA/Ro60 and KCS	0.615	0.963	0.933	0.917
Parotid grayscale ultrasound score and CDS blood grade and anti-SSA/Ro60 and KCS	0.185	0.934	0.869	0.867

CEUS, contrast-enhanced ultrasonography; CDS, color Doppler sonography; MSGB, minor salivary gland biopsy; KCS, keratoconjunctivitis sicca.

To further estimate the contribution of each index to the overall classification ability, an random forest model with runs of cross-validation was conducted. The mean decrease in Gini coefficient of parotid and submaxillary US scores was lower than that of anti-SSA/Ro60 positivity while significantly higher than KCS (**Figures 5B, D**). In addition, CEUS scores also played a role in the model, although the mean decrease in Gini coefficient was lower than KCS. Furthermore, the accuracy of the prediction model was evaluated through calculating the POD value for pSS and demonstrated a significant difference in 1,000 random sampling (p < 0.001; **Figures 5C, E**). To investigate the potential relationship between CEUS scores and grayscale US scores in the submaxillary glands, the correlation analysis demonstrated that the correlation coefficient of submandibular grayscale US scores and CEUS scores was quite low (R = 0.22), although the p-value was 0.001 (**Supplementary Figure S1F**). We further divided the subjects into different subgroups according to the submandibular grayscale US scores (0–4 grades), and a comparison of submandibular CEUS scores between pSS and non-pSS at the identical grayscale US grade was performed. Interestingly, the CEUS scores of pSS were significantly higher than those of non-pSS in grayscale US grades 1 and 2 (p = 0.044, p = 0.040), while they had the same grayscale US grades (p > 0.05) (**Supplementary Figure S1E**).

### Development and Validation of Nomogram for Individual Prediction in Primary Sjögren’s Syndrome

Nomograms were developed to aid in predicting risk of pSS using the four prognostic factors in parotid and submandibular glands (**Figures 5F, G**). The calibration curves exhibited high homogeneity between the prediction results and the observations in the training and validation cohorts, with the C-index of 0.9383 and 0.9088 in the parotid glands, and 0.9680 and 0.9070 in the submandibular glands, respectively (**Supplementary Figures S1I–L**). DCA for the nomogram using pathological examination of salivary, parotid, or submandibular ultrasonographic examination is demonstrated in **Figure 5H**. This revealed that if the threshold probability of a patient was >50%, using submandibular ultrasonographic examination adds more net benefit than using the pathological result of SGs for the prediction of pSS. In addition, when the threshold probability was more than 60%, using parotid ultrasonographic examination adds more net benefit (**Figure 5H**). In addition, CIC analysis for predicting pSS showed that threshold probabilities of >0.5 in submandibular and >0.6 in parotid detection were the most accurate for diagnosing pSS, consistent with the results of DCA (**Supplementary Figures S1G, H**).

## Discussion

Recently, increasing interest has arisen for SGUS as a useful tool for the assessment of major salivary gland involvement in pSS. Major SGs were usually examined by grayscale and color Doppler US in most ultrasonographic evaluations of pSS (34, 35). Despite the effectiveness of grayscale US and color-coded duplex examinations, they can barely assess tissues that are deeply sited or obscured by bone, including the deep lobe of the parotid and deeply sited lymph nodes (36). In addition, its dependence on operator experience and lack of objective quantitative parameters reduce the reliability and stability in detection of pSS. These defects could be well supplemented by CEUS through drawing TICs, and CEUS parameters could qualitatively display the ultrasonographic changes of glands, including TtoP, Grad, Area, PI, Atm, and ID (37).

In this study, we are the first to combine these three ultrasonographic examinations for the detection of pSS. We have described the ultrasonographic signatures in the parotid and submandibular glands from various angles in pSS patients compared with non-pSS individuals. We found typical hypoechoic gland architecture and higher grayscale US scores in both the parotid and submandibular glands of pSS patients, which is consistent with previous studies (38, 39). Notably, there was no significant difference of grayscale US scores between parotid *vs*. submandibular glands in neither pSS nor non-pSS, indicating that the SGUS changes of either parotid or submandibular glands could be used for distinguishing pSS from non-pSS. However, Takagi et al. (40) identified that the diagnostic ability of submandibular US in discriminating pSS was significantly higher than that of parotid US. We also performed the comparison of grayscale US scores between left and right glands, and no significant difference was found. Therefore, unilateral examination is sufficient for the assessment of SG involvement in pSS, as reported previously (41).

CDS examinations were further applied to assess the blood flow velocity of the major SGs in a non-invasive and safe way. The parotid glands of pSS patients displayed more abundant blood intensity than that of non-pSS individuals. This is in accordance with the study by Steiner et al. (42), who used color codex duplex sonography and not only detected marked increased perfusion in pSS but also identified the correlation between blood perfusion and disease activity. A marked hyperemia always accompanies the increase of saliva secretion, which might be interpreted as a compensatory mechanism in pSS patients with xerostomia symptoms (43). Moreover, the hypervascular pattern in SGs appears to be directly associated with conspicuous parenchymal changes, including parenchymal heterogeneity changes and the increase of cyst-like structures, agreeing with the results of grayscale US (44). However, there was no significant difference of CDS grades in the submandibular glands between the pSS and non-pSS cohorts, although CDS grades in the submandibular glands in both groups were higher than that of the parotid glands. This may be due to the significantly abundant blood supply of the submandibular glands, as the parotid glands are traversed vertically by the retromandibular vein and maxillary artery, while the submandibular glands are supplied by the facial artery and vein (36). This results in reduced discriminative ability of CDS in the submandibular glands, as most of the participants showed high baseline levels of CDS blood grades with little distinction. Hence, we infer that abundant blood signals in the submandibular glands might disturb CDS to detect the blood flow changes in pSS, and the parotid glands might be more suitable for discriminating pSS from non-pSS by CDS.

Through conducting quantitative analysis of TIC, CEUS has been widely used in the differential diagnosis of benign and malignant diseases and assessment of treatment responses, such as for lymphoma (45), prostate cancer (46), and thyroid nodules (47). From the TIC, we gain two major types of information, including time-related parameters (Atm and TtoP) and intensity-dependent parameters (PI, Area, and Grad). In this study, CEUS examination indicated that lower levels of CEUS parameters (including Area, Grad, PI, and ID) were found in the submandibular glands of pSS than the non-pSS subgroup, implying potential dispersion dysfunction and reduced glandular activity in pSS patients. Cao et al. (48) found similar CEUS parameter changes in tumors after neoadjuvant chemotherapy, including reduced PI and washing slope. Indeed, peak signal intensity was directly related to blood volume and microbubble concentration (45), while these two elements would be distinctly decreased due to gland dysfunction caused by diseases. In pSS patients, massive structural abnormalities of SGs were demonstrated by histological biopsy, including local vasculitis, germinal center-like structure formation, and lymphocyte infiltration (49, 50). All these structural abnormalities would lead to the decrease of CEUS parameters in pSS patients. To better clarify the most efficient quantitative parameters for CEUS examination, CEUS score was identified in our study based on univariate and multivariate logistic regression analysis, including three essential CEUS parametersGrad, PI, and difference.

A close association between clinical features and US manifestations was revealed by our study. Not only were grayscale US scores significantly positively correlated with SG pathology, anti-SSA/Ro60 positivity, and hyperimmuno-globulinemia, as demonstrated in the study by La Paglia et al. (51), but higher CDS grade scores were also associated with SG pathology positivity. It is noteworthy that, besides significant correlation with the above clinical phenotypes, CEUS scores were also significantly positively associated with ESSDAI grades of pSS, suggesting that CEUS scores might reflect the disease activity of pSS.

As for the discriminative capability for pSS, the mean AUC value of independent grayscale US of submandibular glands was 0.807, while that of combined grayscale US and CEUS scores of submandibular glands, together with anti-SSA/Ro60 and KCS test, independent of SG pathology, was up to 0.963, with a sensitivity of 93.3% and specificity of 91.7%. Meanwhile, combined grayscale US and CDS of parotid glands had a mean AUC value of 0.934, together with anti-SSA/Ro60 and KCS test, with a sensitivity of 86.9% and specificity of 86.7%. Both multi-ultrasonographic combinations showed promising prospects in classification of pSS from non-pSS without performing MSGB. This opens the potential for applying this method to detect pSS patients in clinical practice and providing a new non-invasive tool to map the structure-function change of SGs in this population. Interestingly, we found that submaxillary CEUS scores play a role in discriminating pSS from non-pSS, even in the same grayscale US grades, indicating that CEUS scores could be used as an auxiliary tool to help improve the diagnostic accuracy of pSS by grayscale US as for submandibular glands; however, CDS is a good supplement to grayscale US for the parotid glands.

The nomogram established by incorporating the corresponding submaxillary ultrasonographic signatures and anti-SSA/Ro60 and KCS, except for SG pathology, could be used in clinical practice. This was confirmed by excellent calibration between prediction and observation, which achieved satisfactory discrimination in the training and validation cohorts, with high C-indices, respectively. These results further support that parotid and submaxillary ultrasonographic signatures could be used to facilitate the individualized prediction for the diagnosis of pSS.

There were several limitations of this study. First, all patients were recruited from a single center. Therefore, the study population size was limited. Besides, we used non-pSS as control group rather than healthy control, whose normal labial SGs we were unable to obtain because of ethical concern. However, a small part of the non-pSS, although cannot meet either of the classification criteria at present stage, might develop into pSS, which might potentially influence the classification ability of the model. In addition, the diagnostic value of ultrasonographic signatures for pSS still needs to be validated using multicenter research with a larger patient population. Furthermore, the detailed pathophysiological mechanism of different ultrasonographic signatures of parotid and submaxillary glands in pSS remains to be investigated by further studies.

## Conclusion

In conclusion, we detected and summarized the ultrasonographic signatures of the parotid and submaxillary glands in pSS and evaluated the correlation between ultrasonographic signatures and clinical characteristics. Moreover, we identified compositive CEUS scores to represent the features of CEUS parameters and successfully constructed a combined diagnostic model consisting of submaxillary or parotid gland ultrasonographic signatures and classical clinical characteristics to facilitate accurate individual prediction for pSS. Glandular US examination is a promising alternative to MSGB and a safe and efficient tool for the detection of pSS.

## Data Availability Statement

The original contributions presented in the study are included in the article/**Supplementary Material**. Further inquiries can be directed to the corresponding author.

## Ethics Statement

This study involving human participants was approved by the Ethics Committee of the First Affiliated Hospital of Wenzhou Medical University (2016024), and written informed consent was received from all participants for their enrollment. The patients/participants provided their written informed consent to participate in this study.

## Author Contributions

SX and JJ contributed to ultrasonographical examinations. JL and CZ contributed to the data analysis and drafting of the article. HC and PW contributed to the data analysis. JF and JP contributed to the subject recruitment and clinical information collection. JH contributed to minor salivary gland biopsy. MB and XZ contributed to clinical evaluation. XW contributed to design of the study and revision of the article. All authors contributed to the article and approved the submitted version.

## Funding

This study was supported by the Medical and Health Science and Technology project of Zhejiang Province grants (2021RC090), the Medical and Health Science and Technology project of Zhejiang Province grants (2021KY789), and Wenzhou Science and Technology Project (Y20190680).

## Conflict of Interest

The authors declare that the research was conducted in the absence of any commercial or financial relationships that could be construed as a potential conflict of interest.

## Publisher’s Note

All claims expressed in this article are solely those of the authors and do not necessarily represent those of their affiliated organizations, or those of the publisher, the editors and the reviewers. Any product that may be evaluated in this article, or claim that may be made by its manufacturer, is not guaranteed or endorsed by the publisher.
