# Supplementary figures and images for: Performance Evaluation of Multiple Ultrasonographical Methods for the Detection of Primary Sjögren’s Syndrome
Source: Front Immunol. 2021 Nov 22;12:777322. doi: 10.3389/fimmu.2021.777322 (PMC8646092; doi:10.3389/fimmu.2021.777322)

Supplementary Figure 1

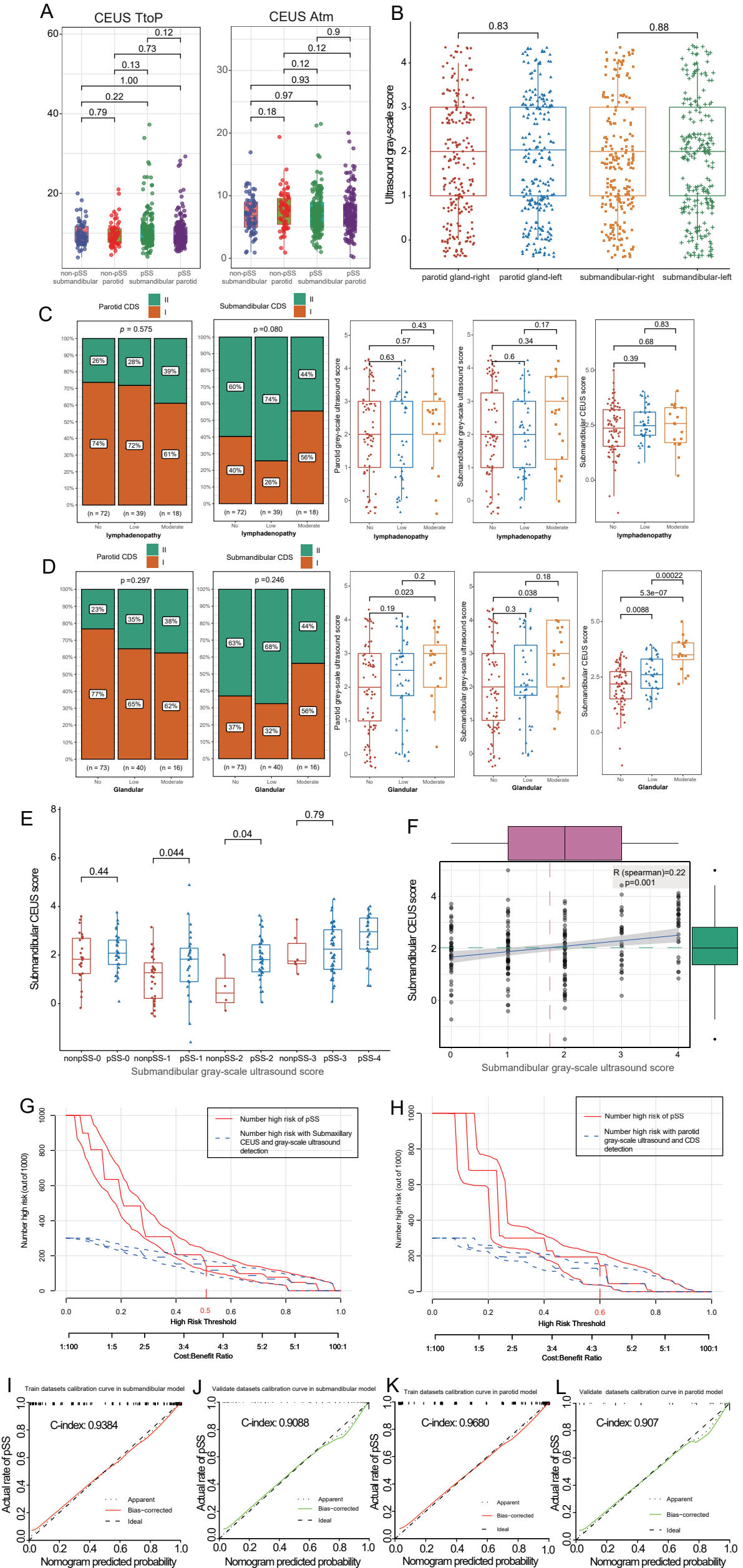

Supplement: Supplementary file 1 [file DataSheet_1.pdf]
