# Supplementary material for: Performance Evaluation of Multiple Ultrasonographical Methods for the Detection of Primary Sjögren’s Syndrome
Source: Front Immunol. 2021 Nov 22;12:777322. doi: 10.3389/fimmu.2021.777322 (PMC8646092; doi:10.3389/fimmu.2021.777322)
Supplement: Supplementary file 2 [file Table_1.docx]

**Supplementary Table1. Univariate and Multivariable analysis of ultrasonic factors in primary Sjogren’s syndrome**

|  | **Univariate analysis** | | |  | | **Multivariable analysis** | | |
| --- | --- | --- | --- | --- | --- | --- | --- | --- |
| **Variable** | **Odds ratio** | **95% confidence interval** | **p. vaule** |  | **Odds ratio** | | **95% confidence interval** | **p. vaule** |
| Parotid TtoP | 1.03 | -0.04~0.12 | 0.418 |  |  | |  |  |
| Parotid Area | 1.00 | 0.00~0.00 | 0.611 |  |  | |  |  |
| Parotid Grad | 0.88 | -0.62~0.38 | 0.625 |  |  | |  |  |
| Parotid Atm | 0.96 | -0.13~0.05 | 0.344 |  |  | |  |  |
| Parotid PI | 0.93 | -0.15~0.01 | 0.110 |  |  | |  |  |
| Parotid ID | 0.97 | -0.13~0.06 | 0.512 |  |  | |  |  |
| Submaxillary TtoP | 1.07 | 0.00~0.15 | 0.081 |  |  | |  |  |
| Submaxillary Area | 1.00 | 0.00~0.00 | 0.004^**^ |  |  | |  |  |
| Submaxillary Grad | 0.65 | -0.83~-0.06 | 0.027^*^ |  | 0.65 | | -0.72~-0.09 | 0.007^**^ |
| Submaxillary Atm | 1.02 | -0.07~0.11 | 0.672 |  |  | |  |  |
| Submaxillary PI | 0.84 | -0.26~-0.09 | <0.001^***^ |  | 0.69 | | -0.47~-0.28 | <0.001^***^ |
| Submaxillary ID | 0.89 | -0.21~-0.03 | 0.007^**^ |  | 1.22 | | 0.11~0.30 | <0.001^***^ |

Note: TtoP; time to peak; Area, area under the curve; Grad, maximum ascending gradient; Atm, arrive time; PI, peak intensity; ID, intensity difference. * p<0.05; ** p<0.01; *** p<0.001.
